# Perspectives for Primary Ciliary Dyskinesia

**DOI:** 10.3390/ijms23084122

**Published:** 2022-04-08

**Authors:** Zuzanna Bukowy-Bieryllo, Michal Witt, Ewa Zietkiewicz

**Affiliations:** Institute of Human Genetics PAS, 60-479 Poznan, Poland; zuzanna.bukowy-bieryllo@igcz.poznan.pl (Z.B.-B.); michal.witt@igcz.poznan.pl (M.W.)

Primary ciliary dyskinesia (PCD) is a ciliopathy caused by genetically determined impairment of motile cilia–organelles present on the surface of many types of cells [1,2,3]. Defects of cilia in the respiratory epithelial cells lead to the impaired mucociliary clearance, resulting in recurrent infections of the upper and lower respiratory tract (sinuses, nose, ears, bronchi, lungs). With time, frequent infections may lead to bronchiectases and impaired pulmonary function, in the most severe cases requiring lung transplantation [2,4]. Defects of motile cilia present in the fallopian tubes epithelium reduces fertility in women, while immotility of sperm tails causes infertility in men [2]. Defective function of motile cilia present in the embryonic node leads to laterality defects in approximately 50% of patients with PCD, manifesting as the inverted positioning of the heart and/or viscera (*situs inversus*); this subtype of PCD is named Kartagener’s syndrome. Depending on the population, PCD occurs in 1 in 10,000 to 1 in 20,000 live births [2].

PCD has been first described in 1936 [5], and the first causative gene has been reported in 1999 [6]. Since that time, PCD has become recognized as a highly heterogeneous disease. To date, over 50 causative genes have been discovered [7,8,9,10,11,12,13,14,15,16,17], with a family and/or population-specific range of mutations found among patients. Despite the high number of genes associated with PCD pathogenesis, the genetic cause of the disease in approximately 30% of patients remains unclear [18]. Therefore, it is important to search for new candidate PCD genes and to investigate their role in motile cilia biology. The Special Issue presents the recent findings related to PCD genetics and molecular mechanisms, as well as the new diagnostics and therapeutic perspectives.

The original article by Sedova et al., describes how pathogenic variants of Nme7 (non-metastatic cells 7, nucleoside diphosphate kinase 7), a protein related to the centrosome function, affect rat embryo development [19]. The Nme7 knock-out rats were generated using CRISPR/Cas9 approach; the homozygous Nme7 deletion was semi-lethal, with the surviving pups presenting sinopulmonary disease, hydrocephalus, *situs inversus totalis* and sterility. Moreover, the reduced number of cilia in the airways and oviducts, but normal architecture of motile cilia were observed; heterozygotes developed normally. The relevance of these findings to human disease remains to be validated, but the paper supports the previously suggested role of Nme7 in ciliogenesis and ciliary transport.

An interesting case of the atypically mild PCD phenotype associated with pathogenic variant of *ODAD1* (*CCDC114*) gene is presented in the original article by Ostrowski et al. [20]. The authors demonstrated that the presence of a homozygous non-canonical splice site mutation resulted in the expression of a truncated ODAD1 (CCDC114) protein, which was assembled into the axonemes, resulting in only partial cilia motility dysfunction. The authors suggested that partial restoration of ciliary function by therapeutic agents could lead to significant improvement of disease symptoms.

The current knowledge on the genotype–phenotype correlations in PCD is presented in the review article by Brennan et al. [21]. Careful examination of patients’ phenotypes combined with the use of multiple diagnostic methods revealed that the genetic heterogeneity of PCD is not only associated with a broad spectrum of the ciliary structure and function defects, but is also reflected in the variable intensity of clinical symptoms, that affects the efficiency of screening tests.

In the original article, Cockx et al., show how careful examination of PCD patients can reveal new clinical symptoms and disclose novel, previously not recognized, molecular defects [22]. The authors found that, in spite of the elevated number of neutrophils present in the peripheral blood of PCD patients, their function was impaired, as seen by the reduced production of reactive oxygen species and a reduced ability to form extracellular pathogen traps (NETs). This was in contrast to patients with other respiratory diseases, such as CF or COPD, where an over-activation of neutrophils relative to controls is observed. The reduced neutrophil activity in patients with PCD may contribute to the recurrence of lung infections.

Cilia are evolutionarily conserved structures, and many cellular and animal models may be used to study the effects of particular gene mutations on cilia function, embryonic development or functioning of body organs. The usefulness of different animal and cellular models for studying PCD-related genes is described in a review article by Niziolek et al. [23]. The authors compared both single-cell models (*Chlamydomonas*, *Trypanosoma*, *Tetrahymena,* and *Paramecium*), and multicellular models - the invertebrate *Schmidtea*, and vertebrates, such as zebrafish, *Xenopus*, and mouse, highlighting their pros and cons, and summarizing experimental data collected using these models.

A detailed analysis of the feasibility of using the zebrafish model in PCD research is presented in the original article by Pinto et al. [24]. Using TEM and ET, the authors characterized the structure of cilia present in two organs present in the zebrafish embryo: the olfactory pit (OP), and the left-right organizer (LRO). Specific differences in the structure of motile cilia present in these organs were demonstrated. Different regions of OP contained motile or immotile cilia, both types with the 9 + 2 structure that is usually associated with motile cilia. The motile cilia in LRO mostly had the 9 + 2 structure. The central pair of microtubules was also commonly observed in some of the motile cilia in LRO, contrary to mouse embryos. The authors conclude that zebrafish is a good model organism for PCD research but investigators need to be aware of the specific physical differences to correctly interpret the analysis results.

The diagnosis of PCD is still difficult due to the variability of clinical manifestations and high genetic heterogeneity of the disease. High-throughput DNA analysis technologies, which generate large amounts of data, must be analyzed in an appropriate manner to ensure that the identified variants are indeed causative for the disease. The article by Števanovic et al., proposes a unique pipeline that uses combination of data available from in silico databases, prediction tools and functional analyses to assess the pathogenic and clinical impact of a large number of genetic variants of unknown significance (VUS) identified in NGS sequencing [25]. The authors positively validated their pipeline by analyzing pathogenic impact of variants identified in both known and candidate PCD genes (*DNAI1* and *SPAG16*, respectively), concluding that application of this approach could lead to a more reliable PCD diagnosis.

So far, no causative drug for PCD has been found and treatment efforts are largely aimed at improving mucociliary clearance and early treatment of bacterial infections of the airway; treatment guidelines are largely based on cystic fibrosis (CF) guidelines. The review article by Paff et al., summarizes the recent clinical trials and several case reports in PCD [26]. In addition, the authors explore potential use of precision medicine approaches, including gene, transcript, or read-through therapies.

## Conclusions

The heterogeneity of PCD makes it a challenging disease to diagnose, and treat. Not all PCD genes have been identified, and causative pathogenic variants are not being found in 30% of patients; moreover, a large proportion of PCD individuals have mutations with the still unconfirmed association with the disease. The increasing access to modern technologies of DNA analysis, the availability of various model organisms for studying the biology of candidate genes and the effect of newly identified mutations, and the increasing understanding of the genotype–phenotype relationship, should increase the efficiency of molecular diagnosis in patients and foster further studies on the practical application of new therapies, to improve the quality of life for PCD patients.
